# Digital twin-based multi-level task rescheduling for robotic assembly line

**DOI:** 10.1038/s41598-023-28630-z

**Published:** 2023-01-31

**Authors:** Bitao Yao, Wenjun Xu, Tong Shen, Xun Ye, Sisi Tian

**Affiliations:** 1 School of Mechanical and Electronic Engineering, Wuhan University of Technology, Wuhan, 430070 China; 2 School of Information Engineering, Wuhan University of Technology, Wuhan, 430070 China; 3 Hubei Key Laboratory of Broadband Wireless Communication and Sensor Networks (Wuhan University of Technology), Wuhan, 430070 China

**Keywords:** Electrical and electronic engineering, Information technology

## Abstract

Assembly is a critical step in the manufacturing process. Robotic assembly technology in automatic production lines has greatly improved the production efficiency. However, in assembly process, dynamic disturbances such as processing time change and advance delivery may occur, which cause the scheduling deviation. Traditional scheduling methods are not sufficient to meet the real-time and adaptive requirements in smart manufacturing. Digital twin (DT) has the characteristics of virtual-reality interaction and real-time mapping. In this paper, we propose a DT-based framework of task rescheduling for robotic assembly line (RAL) and its key methodologies, thus to realize the timely and dynamic adjustment of scheduling plan under uncertain interferences. First, a DT model of RAL task rescheduling composed of physical entity (PE), virtual entity (VE), and virtual-reality interaction mechanism is proposed. Then, a mathematical model is established. By analyzing the adaptive objective thresholds from the perspectives of event trigger and user demand trigger, a DT-driven multi-level (production unit level and line level) rescheduling strategy is proposed. Taking both the computing time and solution quality into consideration, the precedence graph is introduced to propose a rescheduling approach based on an improved discrete fireworks algorithm. Finally, the effectiveness of the proposed model and approach are verified by task scheduling experiments of RAL.

## Introduction

Manufacturing is the pillar of economy of many countries. Smart manufacturing has become the trend of the next industrial revolution where information technology and artificial intelligence (AI) are used to improve the efficiency of manfuacturing^1^. Assembly is a critical step in the manufacturing process. The assembly time accounts for 40–60% of the entire manufacturing time^2^. As the typical manufacturing equipment, industrial robots have been widely used in manufacturing due to their high efficiency and reliability. Robotic assembly has greatly improved the efficiency of product assembly.

Assembly-oriented production scheduling is a decision-making process in which limited assembly resources are matched with assembly tasks under certain sequence and time constraints, so as to optimize the assembly scheduling objective^3,4^. Assembly sequence planning (ASP) is needed for production scheduling^5–8^ and it is an NP-hard problem^9,10^. There are many studies on ASP. For example, in order to obtain the feasible assembly sequence in the shortest time, Zhang et al.^4^ proposed an ASP method based on assembly precedence graph, where a simple firework algorithm was used to minimize the number of changing assembly direction and switching assembly tools. Based on ASP, the production tasks can be scheduled.

Cycle time is a key factor in robotic assembly line (RAL) task scheduling^11^. Failure to deal with uncertain events in assembly process such as machine failure^12^ and arrival of urgent orders^13^ are common dynamic factors that affect assembly efficiency. There are many studies on production rescheduling considering the dynamic factors. Li et al.^14^ studied the dynamic scheduling and due date assignment of one-of-a-kind assembly production when the processing time is uncertain and normally distributed. Liang et al.^15^ proposed a machine failure prediction method based on convolutional neural network, which triggers rescheduling when the machine fails. Considering the uncertain processing time and random machine breakdown, Zheng et al.^16^ proposed a modified master-apprentice evolutionary algorithm to enhance the robustness of the scheduling system. Shahrabi et al.^12^ took random arrival of jobs and machine failures into consideration and used the reinforcement learning method based on Q-learning to determine the time node for rescheduling. Another reinforcement learning method based on dual Q-learning was used to solve the job shop scheduling problem with different task arrival frequencies^17^. To minimize the total delay time under the urgent insertion of new tasks, Luo et al.^13^ used Markov decision process to describe the dynamic flexible job shop problem, where the agent can determine the tasks to be processed at the next moment and the corresponding machine, and learn the most appropriate scheduling rule at each rescheduling point. Zhang et al. considered rescheduling upon disruptions and proposed a hybrid MPGA-CP approach to optimize the makespan, maximum machine workload, and total tardiness^18^. As its capability to cope with uncertainty in a dynamic environment, self-learning capability, and computationally efficient and highly adaptive, reinforcement learning is an emerging technology that has been applied in dynamic task scheduling^19^. Johnson et al. propose a Multi-Agent Reinforcement Learning system for scheduling dynamically arriving assembly jobs in a robot assembly cell^20^.

With the increasing need for individualized products, there are more uncertainty factors and production becomes more complex and dynamic. This put much higher requirements on task scheduling, especially that uncertainty factors will exert a strong influence on the accuracy of workshop scheduling^21^. The interaction between the scheduling model and the physical production line is of significant importance. Many previous task scheduling has not put much attention on this interaction, making it difficult to make effective and accurate response to the uncertainty factors in physical workshop^22^.

CPS integrates the physical system and cyber system. Qiao et al. proposed a closed-loop adaptive scheduling solution based on the Cyber-Physical Production System (CPPS) to deal with the dynamic and uncertain manufacturing environment^23^. Digital Twin (DT), first introduced as “digital equivalent to a physical product” by Michael Grieves at University of Michigan in 2003^24^, is a key technology to realize the interaction of physical space and virtual space^25,26^. Compared with CPS, models play an important role in a DT to help interpret and predict the behavior of the physical space based on various data^27^. DT has attracted extensive research in academia and a wide range of applications in manufacturing, such as digital twin workshop^28^, human–robot collaborative assembly^29^, product lifecycle prediction^30^, product design^31^, and optimization of production scheduling^21^. The production process and status data of physical entity (PE) can be fed back and presented through the virtual entity (VE), while the production plan can be simulated in VE and the behavior of the PE can be intelligently adjusted. Through continuous iterative optimization between VE and PE, DT itself has been continuously evolved and improved. Therefore, it’s suitable to apply DT in RAL task scheduling, where VE is the real representation of PE, to realize scheduling optimization in uncertain environments.

Constructing the digital twin model of research objects is the primary task of digital twin applications. Zhang et al.^32^ constructed a digital twin model of stamping production line based on physical data, which includes the physical model, the 3D virtual model, and the ontology-based information model. Du et al.^33^ constructed a unified model of manufacturing capability and a reconfigurable digital twin model of robotic manufacturing systems. Tao et al.^34^ proposed a digital twin five-dimensional model consisting of physical entity, virtual entity, digital twin data, system services and connections, which is adopted by many researchers^35–37^. Lu and Xu^38^ studied modeling languages such as Semantic Web, OWL, and Jena, regarded resource virtualization as a key enabling technology for creating virtual entities of smart factories. Shi et al.^39^ constructed a DT logic model from the dimensions of geometry, physics, production behavior, and simulation rules for the production line logic simulation, and verified the correctness of the production line design scheme.

Recently, the applications of digital twin in manufacturing bring a chance to reduce the gap between scheduling plan and actual production environment^40^. Zhang et al.^41^ realized further integration of physical and virtual space with the constructed five-dimension digital twin, which provided information for machine availability prediction, disturbance detection and performance evaluation by fusing real and simulated data and triggered rescheduling in time. Fang et al.^42^ realized the real-time mapping and interaction of digital twin based on perception data and monitored abnormal events and proposed an improved multi-objective optimization algorithm to solve the flexible job shop scheduling problem by real-time monitoring abnormal conditions in production. Negri et al.^43^ proposed a scheduling framework that considers equipment health prediction and embedded a field-synchronized equipment health indicator module into the digital twin simulation platform. Later, they considered how to collect and analyze a large amount of field data in real-time through the module in the case of production uncertainty and calculated the failure probability of the current flow shop equipment^44^. Considering the structural heterogeneity of data, Bao et al.^45^ proposed an assembly-oriented part digital twin modeling method where ontology has been used to realize the real-time simulation and adjustment in assembly process. Xu et al.^46^ proposed a digital twin-based industrial cloud robotics framework and the control approaches based on it.

In this paper, we propose a DT-based framework for task rescheduling for RAL. DT enables the bidirectional mapping between the PE and the VE and it provides real time task and equipment condition data for the rescheduling of RAL, thus the dynamics and uncertainties of the physical production line can be considered in time and the scheduling can be accurate. Based on the framework, we also propose an improved discrete fireworks algorithm which saves computing time and makes it possible for real-time rescheduling of RAL, which is vital important for DT. An assembly case is used to verify the real-time capability and the capability to deal with dynamic events of the proposed methods.

## Framework of DT-based task rescheduling for RAL

To implement scheduling in DT, the RAL task scheduling problem is first introduced in this section. Then, a DT-based task rescheduling framework is illustrated. The main components of DT model for constructing a digital twin are presented. DT model is modelled based on PE, VE, and virtual-reality interaction. For convenience, the abbreviations in this paper are listed in Table 1.

**Table 1:** Abbreviations list.

Abbreviation	Description	Abbreviation	Description
DT	Digital twin	IGD	Inverted generational distance
RAL	Robotic assembly line	GA	Genetic algorithm
PE	Physical entity	DABC	Discrete artificial bee colony algorithm
VE	Virtual entity	EDBA	Enhanced discrete bee algorithm
ASP	Assembly sequence planning	IDFA	Improved discrete fireworks algorithm
CPS	Cyber-physical system	ASPM-PG	Assembly subsets prediction method based on precedence graph
CPPS	Cyber-physical production system		

### Problem formulation

The RAL is composed of multiple robotic assembly workstations, where products are assembled in batches. The robots of each assembly workstation perform assembly tasks in parallel at the same time. According to the assembly process, the processing object passes through each workstation in turn while parts are gradually added to the product and the finishing of the final product is at the end of RAL. Before the start of assembly production, ASP is carried out and the assigned tasks are supposed to be completed within the cycle time of RAL. During the assembly process, there may be uncertain events or user demands such as change in processing time, advance delivery, the arrival of emergency orders, etc. Timely rescheduling of the initial scheduling plan can enhance the adaptability of the RAL task scheduling system. To simplify the problem, the constraints and assumptions are set as follows.

1. There is one robot in each workstation of RAL. At a certain time, each robot can process only one task.
2. There is priority relationship among the tasks and one assembly task corresponding to a part. The subsequent tasks can only be processed after the previous task.
3. The assembly process of RAL is simplified as tool change (if necessary), movement and assembly.
4. The working time of each workstation is supposed to be within the cycle time of RAL, and the maximum completion time of the task is supposed to be earlier than the delivery time.

### Framework of DT-based task rescheduling

**Figure 1:**
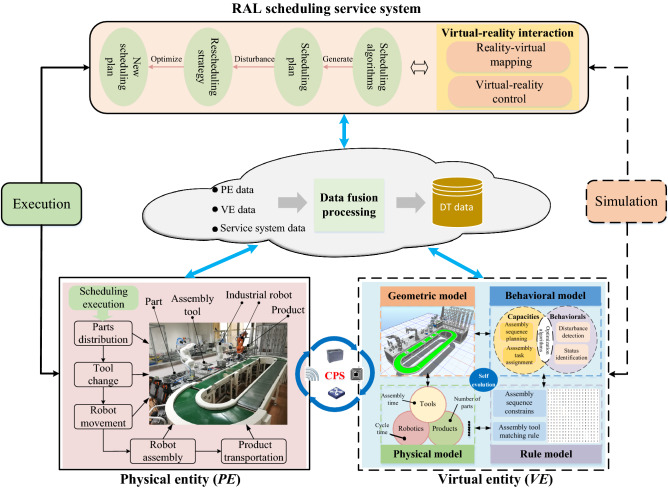
Framework of DT-based task rescheduling.

The DT-based task rescheduling framework is illustrated in Fig. 1. A detailed description is as follows.

- RAL physical entity mainly consists of scheduling resources and the scheduling execution process is decomposed into material delivery, tool change, robot movement, robot assembly, and product transportation.
- RAL virtual entity is responsible for precisely mapping the physical entity, which is integrated and fused by geometric model, physical model, behavioral model and rule model. The geometric model is the high-fidelity visualization of physical entity. The physical model guarantees the geometric model the same properties as physical entity. The behavioral model is the description of static capabilities and dynamic behaviors or motion simulation under the external disturbance. The rule model ensures the accuracy and reliability of the assembly process.
- DT data contain PE data, VE data, and service system data. PE data are condition measurements of physical equipment. VE data are generated in the virtual space. Service system data are generated by the RAL scheduling service system in Fig. 1. Data fusion processing is to obtain effective RAL task scheduling information which can be directly used from massive multi-source assembly process data.
- The RAL task scheduling service system provides the services of generating the initial scheduling plan and the rescheduling plan in the case of disturbance with the support of virtual-reality interaction mechanism.

The PE and VE are interactively connected through the CPS units. Through the assembly sequence constraints in rule model, a feasible assembly sequence is generated in behavioral model. Then, the assembly tasks are assigned to each workstation of RAL reasonably. After simulation in geometric model, the plan is output to physical entity for execution. When dynamic events happened, the parameters of scheduling algorithms can be updated to eliminate the difference between physical and virtual space with analysis of the fused data. Then the simulation is performed again to control the execution process in physical entity. Through continuous iterative optimization, the optimal scheduling of assembly process and the self-evolution of VE comes true.

### RAL physical entity

**Figure 2:**
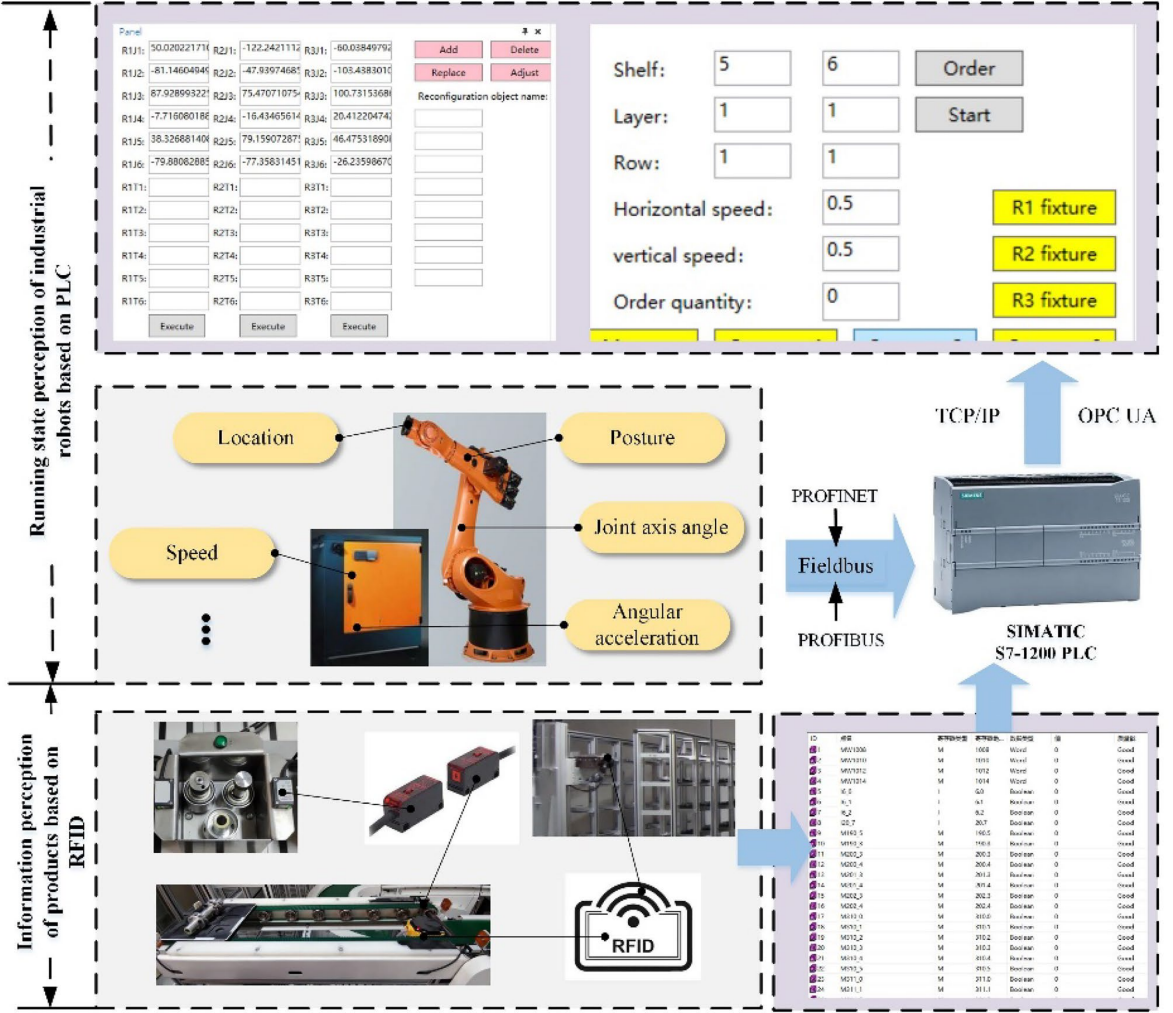
Status perception of PE based on PLC and RFID.

Accurately perceiving the state of production elements in RAL physical entities is the premise of judging uncertain events in assembly process. The status data mainly consists of the operating data of industrial robots and the state information of products, where PLC, RFID sensing technology and other sensors are used to obtain the status data, as shown in Fig. 2.

Take Kuka series industrial robots as an example, its operating data exists in its controller KRC4, which is represented by different internal system variables. The relevant system variables are bound with the digital I/O ports of the controller, and the original system state data are obtained after the data format conversion and then transferred to PLC through the Fieldbus systems, such as PROFINET and PROFIBUS. After data processing, the acquired data are transmitted to the programmable terminal interconnected with PLC through the standard communication protocol, such as TCP/IP and OPC UA. Through the electronic label on the surface of the product and the RFID reader on the platform of the warehouse, real-time information of the product in and out of the warehouse can be obtained. Through the label attached to the surface of the part and the reader at the conveyor belt of the relevant robot workbench and the material box, assembly sequence of the product, robots and tools to assemble the part, as well as the starting or ending time can be obtained.

### RAL virtual entity

Due to the wide variety of RAL production elements and functions, a unified semantic description model of RAL virtual entity is constructed. Ontology, as a knowledge representation method with standardized description framework, can standardize logical description of the research object and is used to describe the RAL virtual entity. In this paper, we define four basic ontologies for RAL virtual entity, namely geometric model ontology, physical model ontology, behavioral model ontology, and rule model ontology.

The formal description of RAL virtual entity is shown in Eq. (1).

1 $$Virtual\_Entity = \{ Geometric\_{\text{M}}odel,Physical\_Model,Behavioral\_Model,Rule\_Model\}$$

The formal description of the geometric model ontology is shown in Eq. (2).

2 $$Geometric\_Model = \{ Shape,Size,Location,Posture,...\}$$

where *Size* represents the length of the joint axis, the width of the conveyor belt, etc. *Location* represents the spatial position of the PE relative to the origin of the world coordinate system, including X, Y and Z parameters. *Posture* represents the rotation angle of PE’s base coordinate system around the X, Y, and Z axis of the world coordinate system, including A, B, and C parameters.

The description of the physical model ontology is shown in Eq. (3).

3 $$Physical\_Model = \left\{ {Robot\_Info,Product\_Info,Tool\_Info,Cycle\_Time,...} \right\}$$

where *Robot_Info* includes robot ID, basic information, capability information, and status information, etc. Robot ID is the unique identifier for each robot. The basic information includes the robot’s specific types. The capability information represents the tools that the robot can use. The status information represents the robot’s three states, namely work, idle and failure. *Product_Info* includes product ID, basic information, and product structure information. Product ID is the unique identifier of the product. The basic product information describes the product name and the total number of parts. The product structure information describes the hierarchy of assemblies. *Tool_Info* indicates the assembly tools used for different parts. *Cycle_Time* represents the start-stop time of RAL, which is critical to the takt time.

The description of the behavioral model ontology is shown in Eq. (4).

4 $$\begin{gathered} Behavioral\_Model = \{ Assembly\_Sequence\_Planning,Assembly\_Task\_Assignment, \hfill \\ Disturbance\_Detection,Status\_Identification,...\} \hfill \\ \end{gathered}$$

where *Assembly_Sequence_Planning* represents the feasible assembly sequence planned according to the spatial interference relationship of parts. *Assembly_Task_Assignment* represents the scheduling plan obtained according to the corresponding relationship between assembly tools and RAL tasks. *Disturbance_Detection* indicates that the dynamic event can be detected through the data exchange and analysis between the perceived data from PE and the historical, simulation and prediction data of VE. *Status_Identification* represents the assembly state at the rescheduling point, mainly including the number of assembled products, the number of semi-finished products, and the number and number of assembled parts of the current assembly product.

The description of the rule model ontology is shown in Eq. (5).

5 $$Rule\_Model = \{ Assembly\_Rule,Matching\_Rule,Accumulated\_Knowledge\}$$

where *Assembly_Rule* refers to the spatial interference or constraint relation of the parts, which is an important reference basis to judge whether the assembly sequence is feasible. *Matching_Rule* describes the corresponding relationship between parts and assembly tools. Generally, one tool can assemble multiple parts with similar appearance and quality. *Accumulated_Knowledge* represents the experience accumulated in the continuous iterative optimization of the VE with the progress of assembly process.

### Virtual-reality interaction

**Figure 3:**
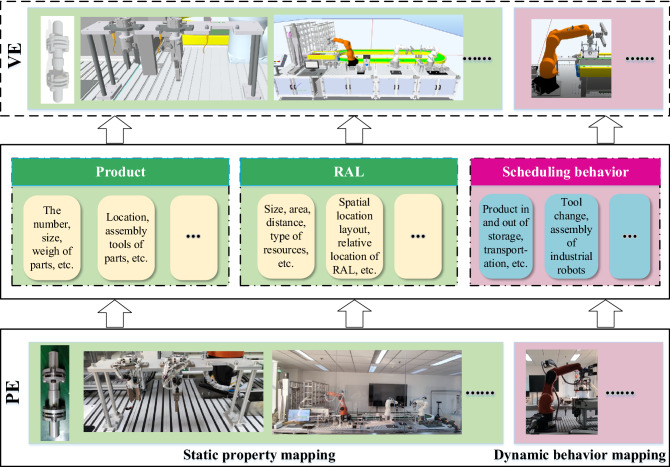
PE-VE mapping.

To realize the interconnection between the PE and VE, and enable the scheduling system to make timely responses and adjustments to uncertain events in the assembly process, the ideal dynamic virtual-real interaction mechanism includes not only virtual-reality mapping mechanism, but also virtual-reality control mechanism which is different from traditional simulation. The PE-VE mapping mechanism is shown in Fig. 3. There is not only the static properties mapping of scheduling resources but also the dynamic logical behavior mapping. The former ensures the consistency of appearance and properties from PE to VE, while the latter ensures that the virtual RAL can synchronize the assembly process of physical RAL.

**Figure 4:**
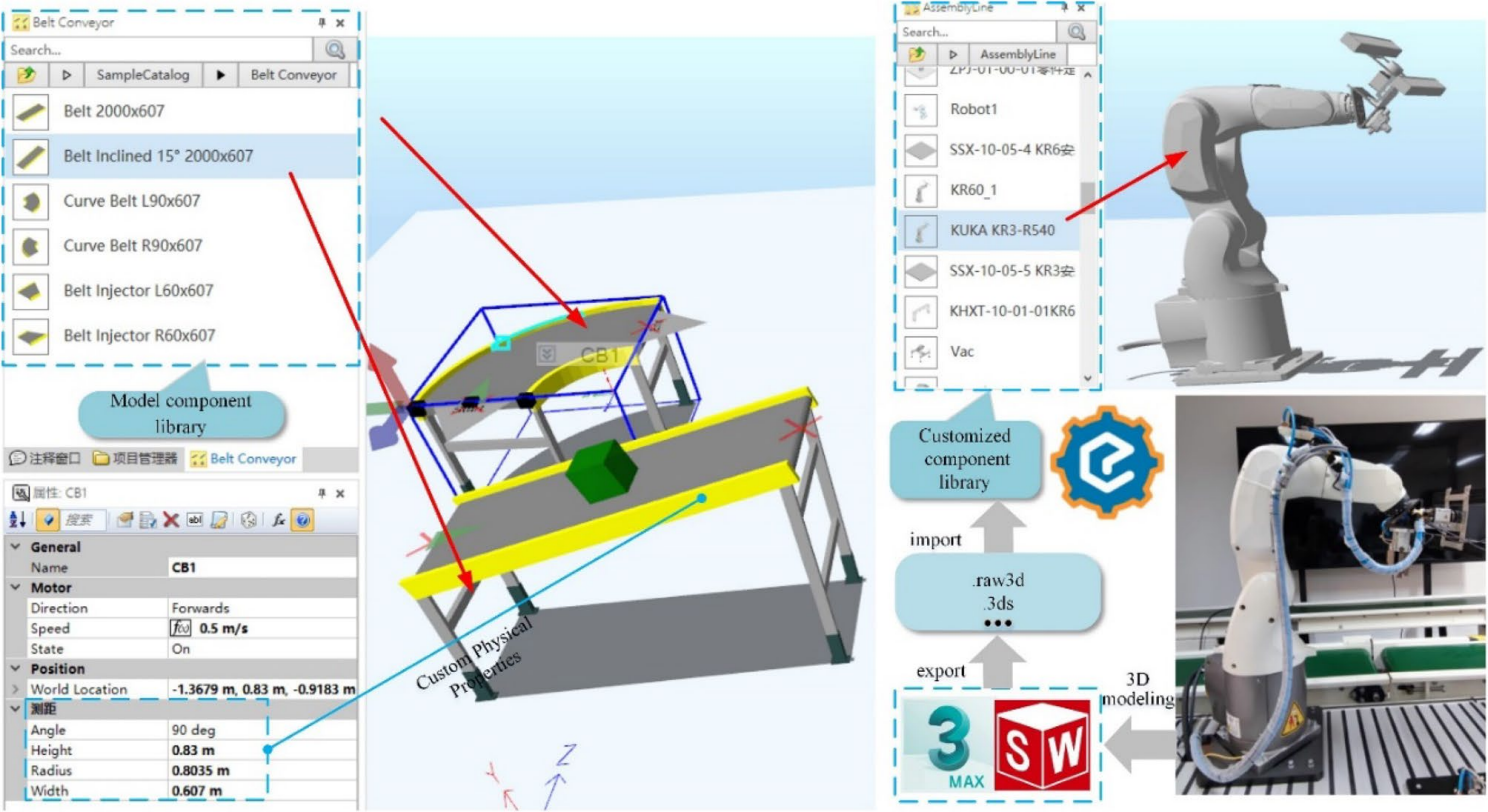
Static property mapping from PE to VE.

For a single scheduling resource, geometric modeling is carried out according to its size, appearance color, location, etc., and it is presented in the form of a visual 3D model. According to the layout of the physical RAL, the relative position between scheduling resources, the visual models are arranged in a corresponding proportion to realize the overall appearance mapping of the RAL. For some general model components, such as conveyor belt and pallets, they can be directly dragged from the preset model component library of Demo3D to the simulation platform and customized properties in the model property window of the simulation platform. For some special customized components, such as the Kuka KR3-R540 industrial robot, the model should be drawn in 3D modeling software first, such as SolidWorks, 3ds Max, etc., and then exported to 3D file formats supported by Demo3D software, such as .raw3d, 0.3ds, .stl, etc. Next import it into the newly created model component library, as shown in Fig. 4.

**Figure 5:**
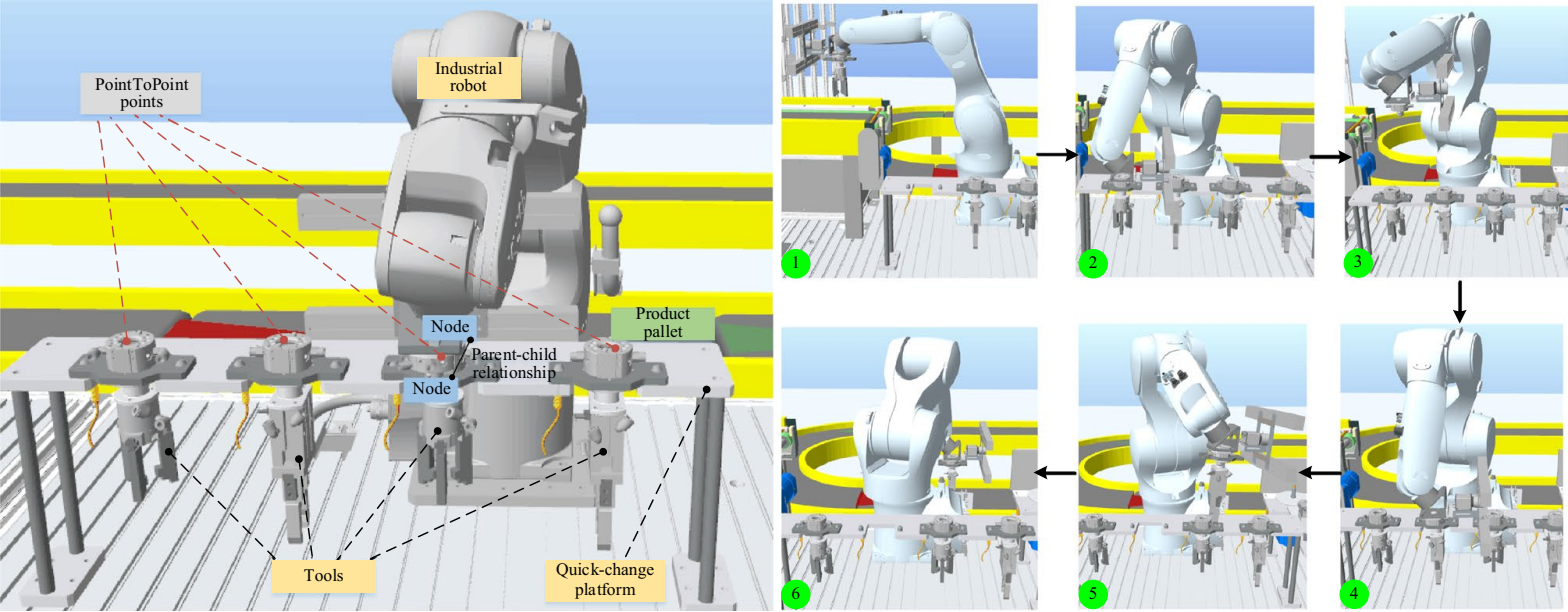
Dynamic behavior mapping from PE to VE.

Dynamic behavior mapping enables that the VE can offline and independently perform the specific assembly processes, i.e. tool changing behavior of robotics. The corresponding PointToPoint points are set according to the spatial position relationship among the nodes, that are the endeffector of the robot, quick-change platform and tools. The parent–child relationship between nodes (different virtual models) is considered, where the child model will move according to the movement of the parent model. As shown in Fig. 5, the tool-changing behavior of Kuka KR6-R700 is illustrated. The initial tool of the industrial robot Kuka KR6-R700 is Gripper-I, as shown in Fig. 5① When it moved to the TeachPonit as shown in Fig. 5②, the parent–child relationship between the tool Gripper-I and the end of the robot is removed. Then the robot has no tool as shown in Fig. 5③. As shown in Fig. 5④, the robot moves to the TeachPonit of spanner-I. The assembly tool is changed and the father-child binding between the end of robot and Spanner-I is built as shown in Fig. 5⑤. Then the robot is ready for the next assembly process as shown in Fig. 5⑥.

**Figure 6:**
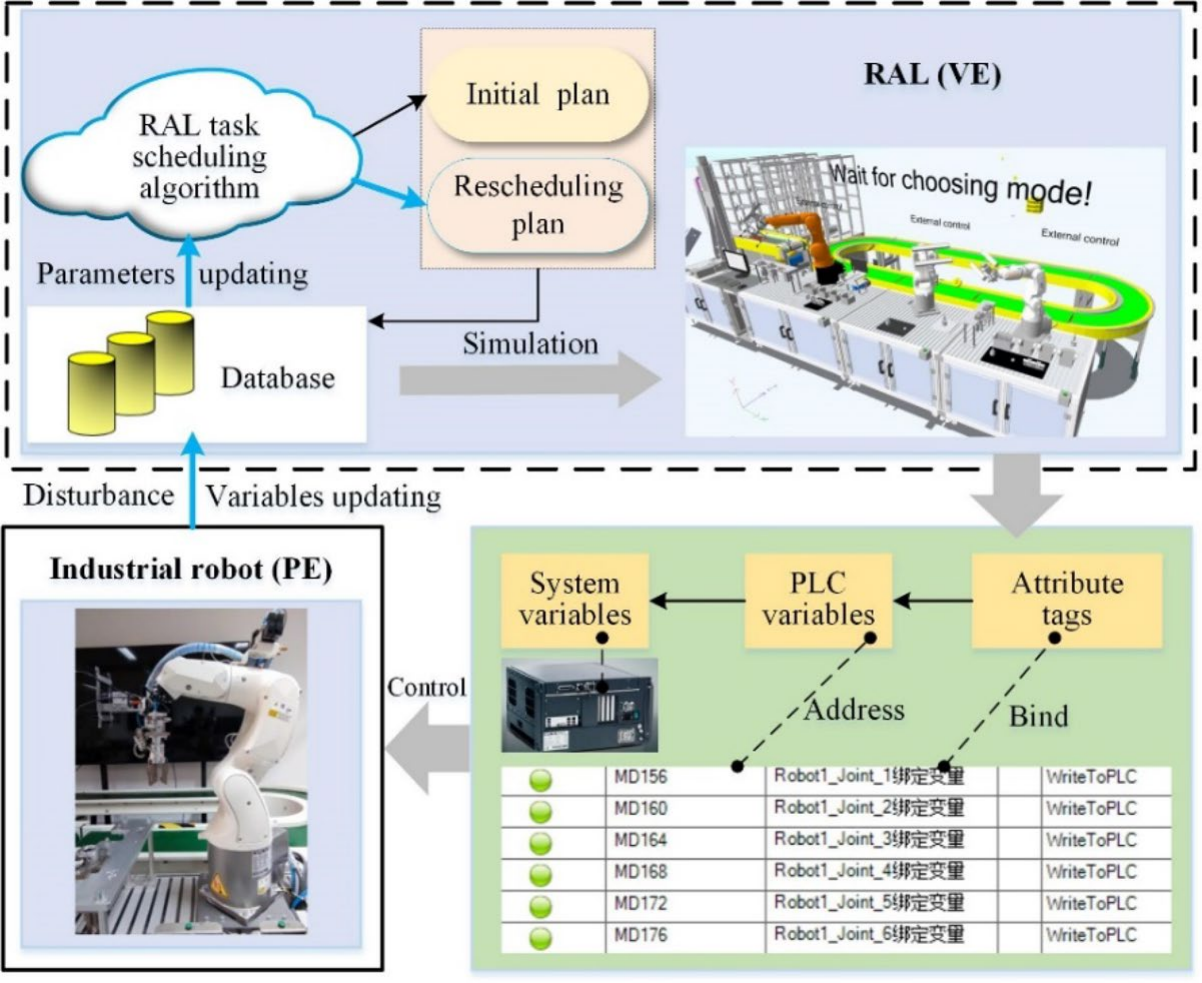
VE-PE control mechanism.

In traditional simulation, the virtual models of the physical entity are used offline to simulate the physical entity. The virtual models are static and lack dynamic interaction with the physical entity. However, DT not only emphasizes the mapping from real to virtual but also the control from virtual to real. The VE-PE control mechanism is shown in Fig. 6. In the RAL assembly process, the state perception data of disturbance are transmitted to the database to update relative state variables. Then the parameters of RAL task scheduling algorithm are updated and a new scheduling plan is generated by monitoring the key scheduling information changes in the database. At the same time, the customized attribute tags of the virtual robot in the simulation platform are bound to the control variables of the geometric model of VE. After completing the format conversion and address mapping with the PLC intermediate variables through the hardware communication interface, the PLC variables are mapped one by one with the system variables of the physical industrial robot through the field bus systems, to realize the transmission of the joint angle value and other state variables from VE to PE and drive the physical robot to move synchronously with the virtual robot.

**Figure 7:**
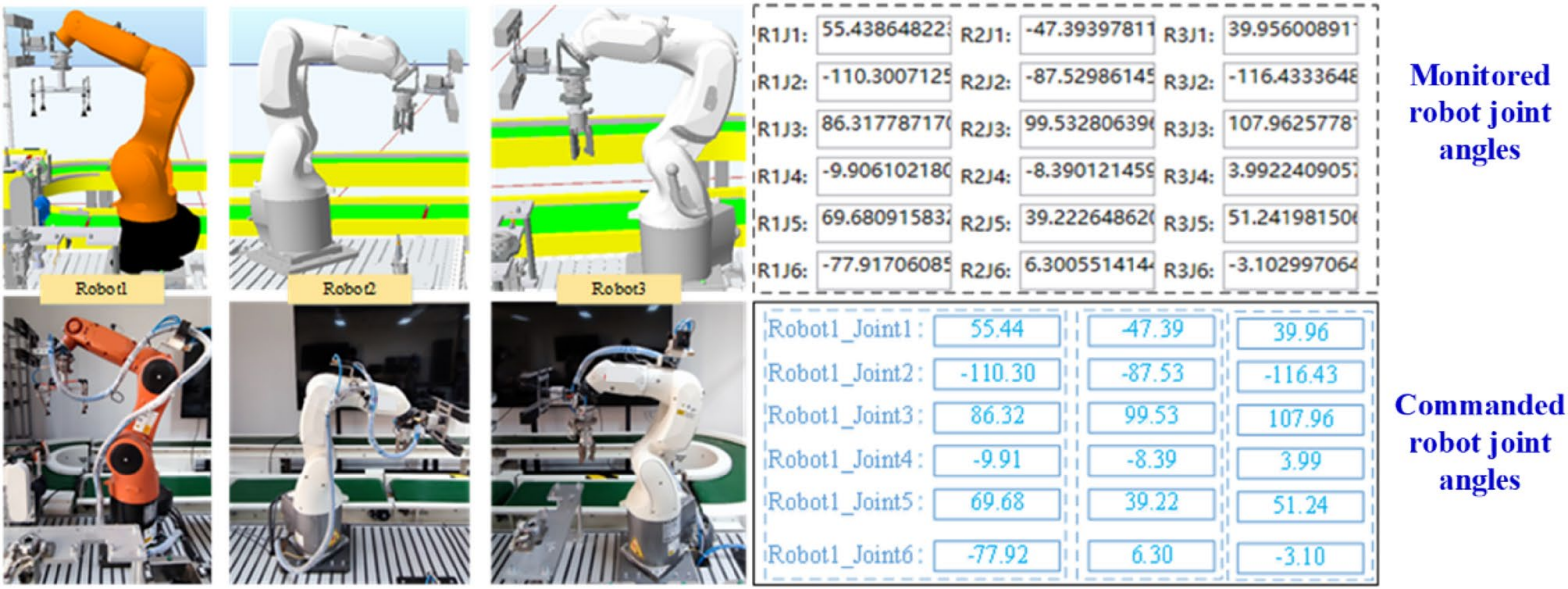
Interactive movement control between VE and PE.

The interaction movement control between VE and PE is shown in Fig. 7. The commanded joint angles of the robots can be input in the human–machine interface and transmitted to the physical robots through the PLC control system to drive them to move synchronously. Then the real angles of robots are monitored and feedback to the human–machine interface. Therefore, the mutual interaction between the VE and PE is realized, and the VE can be updated with the real state of the PE.

**Figure 8:**
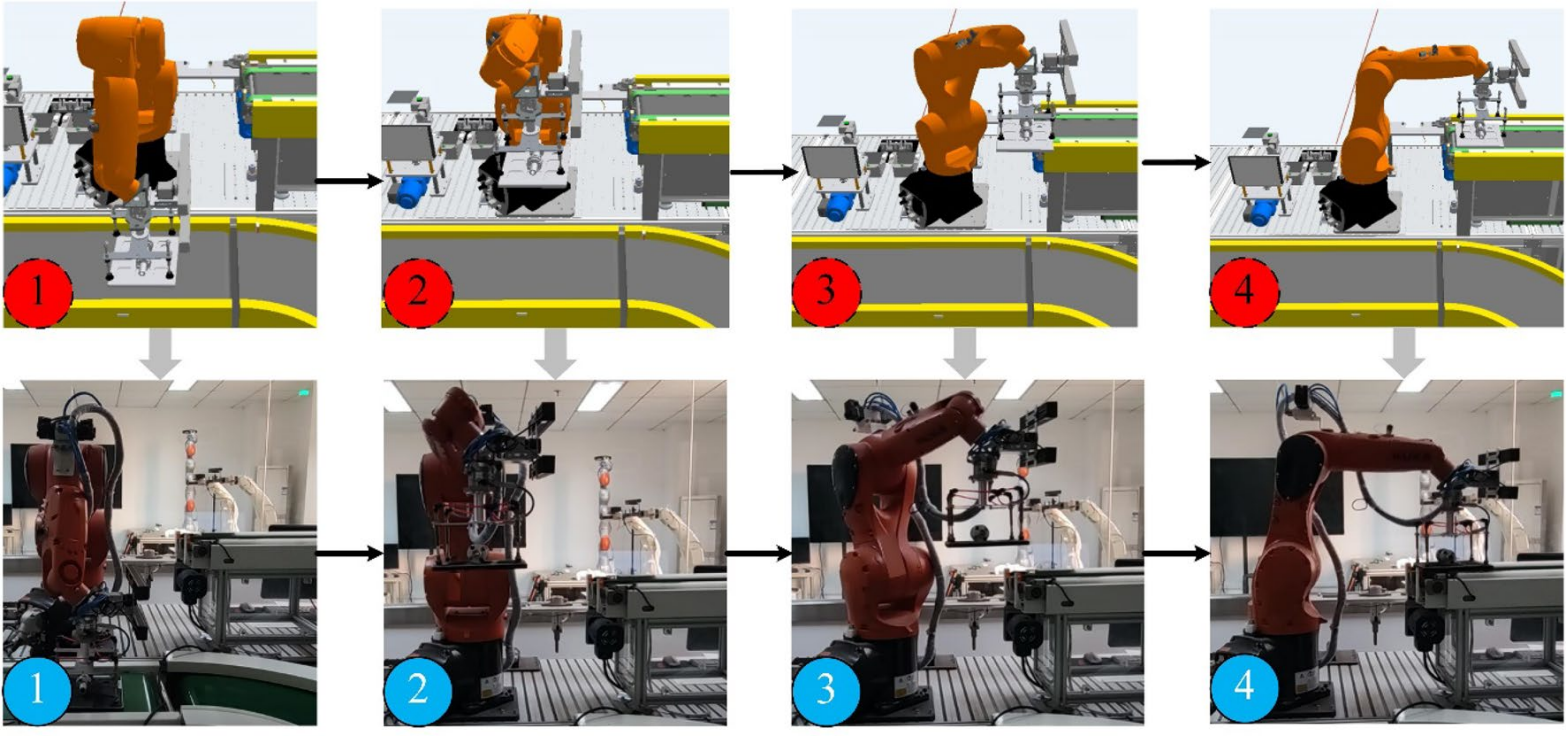
Product warehousing process from VE to PE.

Product warehousing is to transport the assembled products on the conveyor belt to the warehouse’s inbound and outbound platform. The handling path of the industrial robot can be decomposed into these steps, grab the pallet on the conveyor belt, move to the conveyor belt of the inbound and outbound platform and loosen the pallet. The product warehousing process from VE to PE is shown in Fig. 8.

## Rescheduling strategy of RAL tasks based on DT

To solve the RAL task scheduling problem, the mathematical model is illustrated first in this section. Then, the rescheduling strategy which has an adaptive target threshold, considers dynamic events and user demand, and has low computing time is discussed.

### Mathematical model for rescheduling

The symbols used in the mathematical model of RAL task scheduling are list in Table 2.

**Table 2:** List of symbols.

Symbol	Description	Symbol	Description
m	Total workstations	$t_{m}$	Moving time between assembly points
n	Total number of parts of product	$t_{b}$	Basic assembly time of $task_{i}$
N	Total number of products	$n_{i}$	Total number of tasks assigned to $S_{i}$
$S_{i}$	Number of workstations, $i = 1,2,...,m$	$T_{i}$	Working time of $S_{i}$
$R_{i}$	Number of robots, $i = 1,2,...,m$	$Tool_{r}$	Assembly tools available for $R_{r}$
$P_{i}$	Number of parts, $i = 1,2,...,n$	$tool_{ir}$	Tool $R_{r}$ used to complete $task_{i}$
$task_{i}$	Task corresponding to $P_{i}$, $i = 1,2,...,n$	$T_{s}$	Total working time
$t_{ir}$	Total assembly time $R_{r}$ takes to complete $task_{i}$	$L$	Load balancing
$t_{i}$	Completion time of $task_{i}$	$T_{s}^{\prime }$	Total working time after disturbance
DL	Delivery time	$L^{\prime}$	Load balancing after disturbance
$T_{c}$	Cycle time of RAL	$\Delta L_{0}$	Initial threshold of load balancing
$\Delta T_{s0}$	Initial threshold of total working time	$L_{DT}$	Virtual load balancing
$T_{sDT}$	Virtual total working time	$\Delta L$	Threshold of load balancing
$\Delta T_{s}$	Threshold of total working time	$f_{1}$	Objective function of total working time
$t_{t}$	Tool switching time	$f_{2}$	Objective function of load balancing

The objective of assembly sequence planning here represents the total working time for a single robot to complete the assembly tasks, which consists of the tool switching time $t_{t}$, the moving time between assembly points $t_{m}$ and the basic assembly time $t_{b}$. The basic assembly time of part $P_{i}$, $t_{b} (P_{i} )$ is a constant. The tool switching time between the part $P_{i}$ and the part $P_{i + 1}$, $t_{t} \left( {P_{i} , \, P_{i + 1} } \right)$ is shown in Eq. (6). The moving time between assembly points, $t_{m} \left( {P_{i} , \, P_{i + 1} } \right)$ is obtained by dividing the distance between the part $P_{i}$ and the part $P_{i + 1}$ by the moving speed of the robot end-effector. The total working time mentioned above, $T_{\sin }$ is the sum of the time to complete the *n* tasks, as shown in Eq. (7).

6 $$t_{t} (p_{i} ,p_{i + 1} ) = \left\{ \begin{gathered} 0{\text{ tool has not changed}} \hfill \\ 1{\text{ tool size changed}} \hfill \\ 2{\text{ tool type changed}} \hfill \\ \end{gathered} \right.$$

7 $$T_{\sin } = \sum\limits_{i = 1}^{n - 1} {t_{t} (P_{i} ,P_{i + 1} ) + } \sum\limits_{i = 1}^{n - 1} {t_{m} (P_{i} ,P_{i + 1} )} + \sum\limits_{i = 1}^{n} {t_{b} (P_{i} )}$$

The objective function of RAL task scheduling are listed as follows:

1. The total working time of *m* robots in RAL: For workstation $S_{i}$, its working time, $T_{i}$ is the sum of tool switching time, moving time between assembly points and basic assembly time spent to complete the assigned $n_{i}$ assembly tasks, $1 < n_{i} < n$, as shown in Eq. (8). The objective is to minimize the total working time $T_{s}$, as shown in Eq. (9).
  8 $$T_{i} = \sum\limits_{i = 1}^{{n_{i} - 1}} {t_{t} (P_{i} ,P_{i + 1} ) + } \sum\limits_{i = 1}^{{n_{i} - 1}} {t_{m} (P_{i} ,P_{i + 1} )} + \sum\limits_{i = 1}^{{n_{i} }} {t_{b} (P_{i} )}$$
  9 $$\min f_{1} = \min T_{s} = \min \sum\limits_{i = 1}^{m} {T_{i} }$$
2. The load balancing between *m* robots in RAL: In the assembly process, it is necessary to make the working time of each workstation similar and within the cycle time of RAL, so as to reduce the idle time of robots and avoid resulting in the waste of energy consumption. Minimize the load balancing, that is, minimize the sum of squares of differences between the working time of each workstation and the cycle time, as shown in Eq. (10).
  10 $$\min f_{2} = \min L = \min \sqrt {\sum\limits_{i = 1}^{m} {(T_{i} - T_{c} )^{2} } }$$

The constraints are as follows:

11 $$tool_{ir} \in Tool_{r}$$

12 $$t_{i- 1} \le t_{ir} - t_{(i - 1)r}$$

13 $$\sum\limits_{i = 1}^{m} {n_{i} } = n$$

14 $$T_{i} \le T_{c}$$

15 $$N \cdot T_{c} \le DL$$

where Eq. (11) is the assembly tool constraint. Different tools are suitable for different types of assembly tasks. Equation (12) represents the constraint of assembly process. There is a precedence relationship between two successive tasks, and the latter task on the same robot can only be started after the completion of the previous task. Equation (14) indicates that the working time of each workstation should be within the cycle time to ensure the stable running of RAL. Equation (15) represents the constraint of delivery time.

### Rescheduling based on adaptive target threshold

The rescheduling strategy and algorithm consist of an adaptive target threshold, a multi-level (that is production unit level and line level) rescheduling strategy, and the discrete fireworks algorithm with a precedence graph to solve the RAL task scheduling problem.

In dynamic scheduling problems, frequent rescheduling for emergencies adds a burden to the RAL task scheduling system resource allocation process and additional time and cost. In the rescheduling method based on the target threshold, the impact of the current disturbance on the target performance determines whether to reschedule. If it exceeds the threshold, rescheduling is carried out.

The threshold with a fixed value will cause cumulative error as time goes by. The virtual RAL is a faithful mapping of the physical RAL. The virtual data can be regarded as an approximate representation of the physical data, which can be obtained after the process of task scheduling plan simulated in virtual RAL. Due to the communication delay of database interface communication and virtual simulation optimization, the virtual data is generally slightly larger than the data value assumed in the optimization algorithm. In the rescheduling method based on the fixed value of the threshold, the target threshold is the original scheduling target value added with the fixed deviation that the system can tolerate. While the adaptive target threshold in DT can be regarded as the difference between the virtual scheduling target and the target in the initial scheduling plan. At the same time, the updated rescheduling plan becomes the new “initial scheduling plan”, and the updated target threshold becomes the new “initial target threshold”. With the continuous evolution of DT, the scheduling deviation that the RAL task scheduling system can tolerate and adaptability will also be simultaneously improved, changing in a direction closer to the actual manufacturing environment.

If the difference between the total working time after disturbance and the initial total working time satisfies Eq. (16), rescheduling is required, where the threshold of the total assembly time is the initial target threshold added with the difference between the virtual total working time and the initial total working time, as shown in Eq. (18). Similarly, if the difference between the load balancing after disturbance and the initial load balancing satisfies Eq. (17), rescheduling is performed, and the updated load balancing threshold is shown in Eq. (19).

16 $$T_{s}^{\prime } - T_{s} > \Delta T_{s}$$

17 $$L^{\prime} - L > \Delta L$$

18 $$\Delta T_{s} = \Delta T_{s0} + T_{sDT} - T_{s}$$

19 $$\Delta L = \Delta L_{0} + L_{DT} - L$$

### Multi-level rescheduling strategy

In manufacturing systems with a similar structure to RAL, there are some units with different functions that operate cooperatively with each other to ensure efficient operation of the system. The efficiency will be affected if the upper-level production line is required to make response to the disturbance. To reduce the burden, the single assembly workstation of RAL can carry out the decision first. If it cannot provide a scheduling plan that meets the requirements, the production line will carry out the scheduling decision. The unit-level rescheduling strategy is that the partial assembly sequence assigned to the disturbed workstation will be adjusted to optimize the scheduling objective, if more than one workstation is disturbed, each disturbed workstation needs to be adjusted individually. If the optimized plan still does not meet the requirements, the production line-level rescheduling strategy will be carried out, which is the adjustment of the entire assembly sequence and the reassignment of all the tasks.

The uncertain disturbances can be broadly divided into two categories: user demand and dynamic events. If it is a dynamic event and the disturbance it brings doesn’t cause the scheduling objective to exceed the target threshold, continue executing the original scheduling plan. If yes, the production unit and line level rescheduling strategy are carried out. If it is user demand, which is generally related to the order, such as advance delivery, order insertion, etc., the rescheduling optimization is performed directly. The generated new rescheduling plan becomes the new “initial scheduling plan”, and the simulation process comes again to form a closed loop of interactive iteration between the PE and the VE.

### Fireworks algorithm with precedence graph

ASP is an NP-hard problem. To obtain a scheduling plan with high solution quality in the shortest possible computing time can meet the requirements of near real-time scheduling in DT. The Fireworks algorithm (FA) with a low computing time has been successfully applied to ASP, which mainly consists of three steps: initialization, explosion and selection and the final optimization single objective must be consistent with the objective of the subsequence^4^. Also, the precedence graph has been applied, which is a typical expression of assembly interference constraint in product assembly process.

To solve the multi-objective RAL task scheduling problem, Pareto sorting is introduced and the improved discrete fireworks algorithm (IDFA) is proposed. There are two important parameters, namely the population of the fireworks and the number of sparks produced by the firework explosion in FA. The number of sparks determines the local search ability of different fireworks. The brighter the firework, that is, the higher the fitness value, the more sparks it explodes, and vice versa. If a firework generates too many sparks and the population is small, the potential good solutions may not be found and the probability of getting trapped in local optima may be raised. Therefore, the adaptive spark number is proposed, as shown in Eq. (20).

20 $$SP_{i} = NS \cdot \frac{{f_{\max } - f_{i} + \varepsilon }}{{\sum\limits_{j = 1}^{Np} {(f_{\max } - f_{j} ) + \varepsilon } }}$$

In a minimization optimization problem, the number of sparks generated by the fireworks *i* is $SP_{i}$, $NS$ is a constant, $Np$ is the population of fireworks, $\varepsilon$ is a minimum positive number.

**Figure 9:**
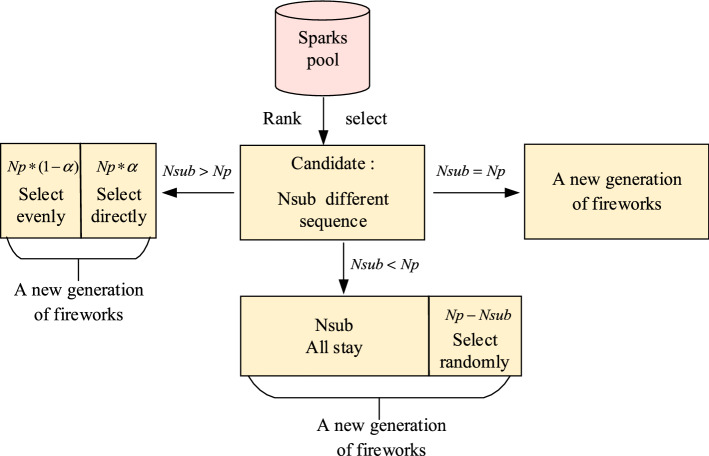
Improved selection strategy.

In the process of selecting sparks from the sparks pool to become a new generation of fireworks, if only the top sparks are selected, there will likely be many individuals with the same sequence, leading to a decrease of population diversity, and thus raises the probability of getting trapped in local optima. Therefore, the improved selection strategy by selecting both top-ranked individuals and some less-excellent individuals is proposed, as shown in Fig. 9. The process is as follows:

- *Step 1*: Sort the sparks individuals in the sparks pool according to the fitness value, and select $N_{sub}$ different sequences as the candidate individuals.
- *Step 2*: If the candidates are smaller than the population, that is $N_{sub} < Np$, then all the $N_{sub}$ candidates will be selected, and the rest will be randomly selected from the candidates. If $N_{sub} = Np$, the candidates just become the new generation of fireworks.
- *Step 3*: If $N_{sub} > Np$, select the top $Np * \alpha$ ($\alpha$ is a coefficient between 0 and 1) individuals, and remove the selected individuals from the candidates. The remaining $Np * (1 - \alpha )$ individuals will be evenly selected from the updated candidates, to form a new generation of fireworks.

The pseudo code of RAL task scheduling based on IDFA is shown in Table 3.

**Table 3:**
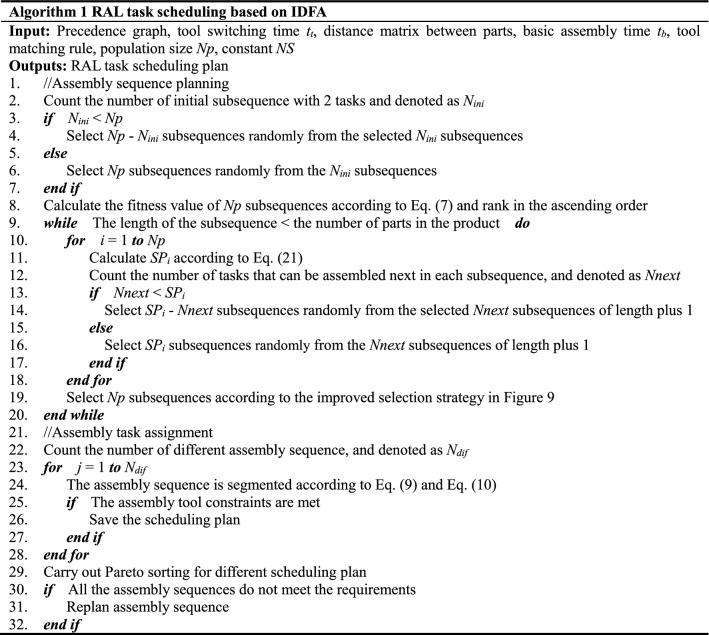
Pseudo code of IDFA.

## Case study and analysis

**Figure 10:**
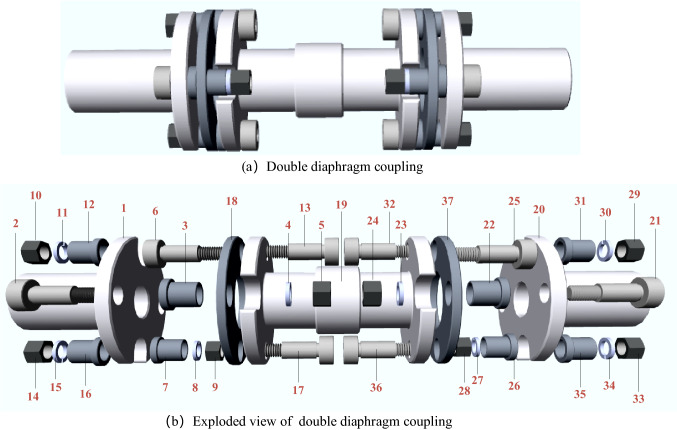
Double diaphragm coupling and the exploded view.

**Figure 11:**
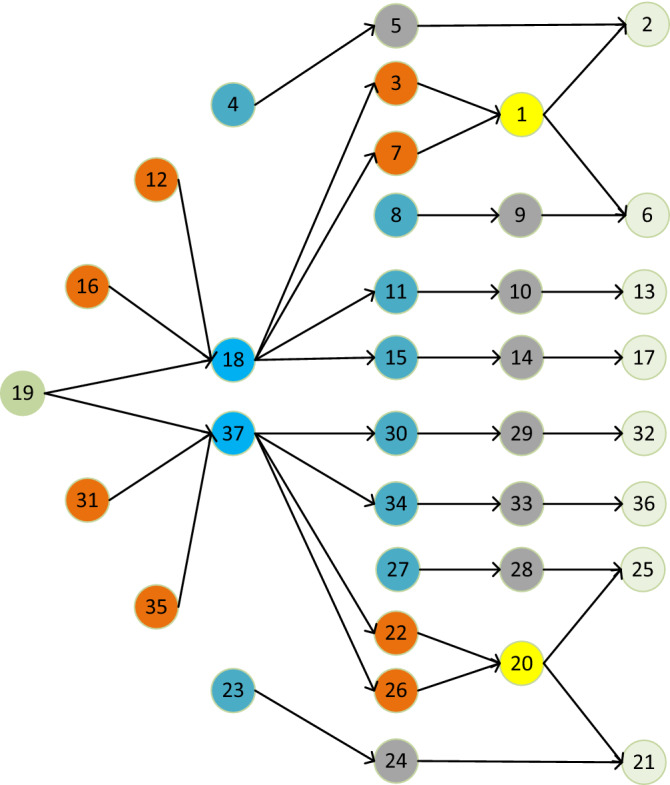
Precedence graph of the product.

To verify the real-time capability which is vitally important for DT, a double diaphragm coupling with 37 parts is used as shown in Fig. 10. The double diaphragm couplings are assembled in batches and the robots work periodically. Figure 11 shows the precedence graph, which can be divided into different types of parts. The same color indicates parts of the same type. The related information of all the parts and tool information of RAL workstations are listed in Tables 4 and 5. The main parameters of the IDFA algorithm are shown in Table 6.

**Table 4:** Information of parts.

Part	Tools	Location (mm)	Basic assembly time (s)
1	Gripper-I	(422, 500, − 524.9)	1
2	spanner-I	(452, 507.3, − 540.8)	4
3	Gripper-II	(462, 507.3, − 540.8)	2
4	Gripper-II	(474.3, 507.3, − 540.8)	3
5	spanner-II	(479.9, 507.3, − 540.8)	5
6	spanner-I	(452, 492.7, − 509)	4
7	Gripper-II	(462, 492.7, − 509)	2
8	Gripper-II	(474.3, 492.7, − 509)	3
9	spanner-II	(479.9, 492.7, − 509)	5
10	spanner-II	(457.7, 515.9, − 517.6)	5
11	Gripper-II	(459, 515.9, − 517.6)	3
12	Gripper-II	(470, 515.9, − 517.6)	2
13	spanner-I	(480, 515.9, − 517.6)	4
14	spanner-II	(457.7, 484.1, − 532.2)	5
15	Gripper-II	(459, 484.1, − 532.2)	3
16	Gripper-II	(470, 484.1, − 532.2)	2
17	spanner-I	(480, 484.1, − 532.2)	4
18	Gripper-I	(467.5, 500, − 524.9)	1
19	Gripper-I	(470, 500, − 524.9)	1
…	…	…	…
31	Gripper-II	(530, 515.9, − 517.6)	2
32	spanner-I	(520, 515.9, − 517.6)	4
33	spanner-II	(547.9, 484.1, − 532.2)	5
34	Gripper-II	(542.3, 484.1, − 532.2)	3
35	Gripper-II	(530, 484.1, − 532.2)	2
36	spanner-I	(520, 484.1, − 532.2)	4
37	Gripper-I	(532.5, 500, − 524.9)	1

**Table 5:** Capability of RAL workstations.

Workstations	Station1	Station2	Station3	Station4	Station5
Tools	Gripper-I/II	Gripper-I/II Spanner-II	Gripper-I Spanner-I/II	Spanner-I/II	Gripper-I Spanner-I/II

**Table 6:** Main algorithm parameters.

Parameters	Value
Population size *Np*	{20,30,40,50,60}
Constant *NS*	300

For multi-objective RAL task scheduling problems, the objectives are shown in Eqs. (9) and (10). $f_{1}$ is the total working time and $f_{2}$ is the load balancing. To verify the effectiveness of the proposed IDFA algorithm, the computing time and solution quality of IDFA were compared with genetic algorithm (GA)^47^, discrete artificial bee colony algorithm (DABC)^48^, enhanced discrete bee algorithm (EDBA)^49^ and assembly subsets prediction method based on precedence graph (ASPM-PG)^4^. The variation probability of GA is 0.1 and crossover probability is 1. The maximum number of iterations of GA, DABC and EDBA is 300. In EDBA, the values of the elite bees, the selected bees, the follower bees led by the elite bee and the follower bees led by the selected bee are 2, 8, 4, 2 respectively. The population size ranged from 20 to 60, and the step size was 10. Under each parameter condition, 20 running experiments are carried out.

**Figure 12:**
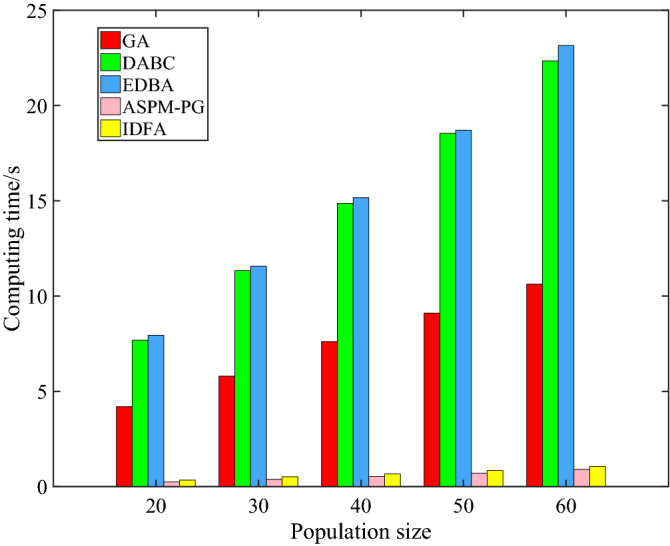
Comparison of average computing time of five algorithms in different populations.

In Table 7, the common parameter, that is the population size, of the 5 algorithms is 30. In terms of solution quality, the two objectives of IDFA algorithm are better than other algorithms. In terms of computing time, IDFA is far lower than GA, DABC and EDBA, although it is slightly higher than ASPM-PG. That is because of the addition of adaptive sparks number and improved selection strategy, but the solution quality is higher than ASPM-PG. In other words, IDFA sacrifices less time to obtain a solution of higher quality. The computing time directly determines the feasibility of the RAL task rescheduling strategy based on DT, and the time delay between getting the scheduling or rescheduling plan in VE and executing in PE determines whether it is near real-time scheduling. The ultimate performance of IDFA in computing time is shown in Fig. 12. the computing time of IDFA is approximately within seconds, while the computing time of GA, DABC and EDBA is approximately at the level of ten seconds, which is unacceptable for DT.

**Table 7:** Performance analysis of 5 algorithms. $f_{1}$ is the total working time (s) and $f_{2}$ is the load balancing (s).

Algorithms	Assembly sequence	*f*_1_ (s)	*f*_2_ (s)	Computing time (s)
GA	8-12-31-27-35-23-24-28-16-4-5-19-37-22-34-26-20-30-29 -21-33-25-32-36-9-18-3-11-7-15-14-1-6-2-10-13-17	230.1919	10.9752	6.7127
DABC	12-31-23-35-27-28-24-16-19 -18-15-4-3-11-7-8-9-1-6-10 -13-37-22-34-5-2-14-17-26 -30-20-33-21-29-25-32-36	230.5780	13.5870	13.4361
EDBA	16-12-4-19-18-8-27-31-23-2 -35-37-26-28-30-22-34-33-2 -29-25-21-32-36-11-3-15-14 -5-10-13-17-9-7-1-6-2	228.5028	11.7398	14.0185
ASPM-PG	16-8-12-19-18-4-3-11-7-15 -14-17-35-31-37-27-26-30-22 -23-34-33-28-29-20-25-36-24 -21-32-5-10-1-2-13-9-6	229.2138	8.1431	0.4418
IDFA	12-16-19-18-7-8-15-11-3-4 -31-35-37-27-26-34-23-22-30-29-24-32-28-33-20-21-25-36-5-10-13-9-14-1-2-6-17	227.3279	6.8161	0.7766

Although the computing time is critical for rescheduling based on DT, the solution quality cannot be ignored. The comprehensive performance evaluation index, IGD (Inverted generational distance), was adopted to analyze the convergence and diversity of IDFA. IGD needs the optimal Pareto solution set or the real Pareto frontier as the reference set. In this paper, five algorithms are independently run 20 times under each population parameter condition. The Pareto solution set of different algorithms is selected as the candidate set first, and then remove the duplicate one and carry out Pareto sorting to obtain the reference set. The smaller the IGD value, the higher the convergence and diversity. The convergence is evaluated by the proximity between the Pareto solution set to be evaluated and the reference set, and the diversity is evaluated by the distribution performance of the Pareto solution set to be evaluated.

**Figure 13:**
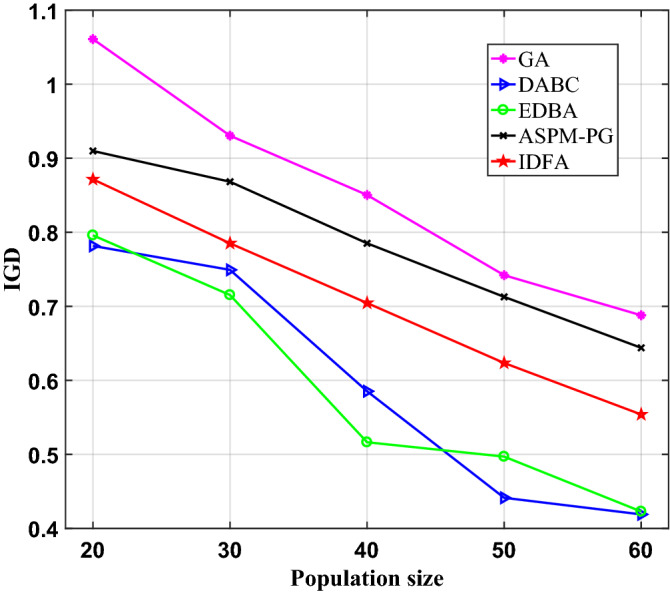
IGD of five algorithms in different populations.

As shown in Fig. 13, the IGD of the five algorithms all decrease with the increase of population size. The IGD of IDFA is smaller than that of GA and ASPM-PG under different population sizes, and is larger than that of DABC and EDBA. The convergence and diversity of IDFA are stronger than that of GA and ASPM-PG, but weaker than that of DABC and EDBA. Compared with ASPM-PG, IDFA only sacrifices less time in exchange for higher quality solutions. Considering that control mechanism from the VE to PE and real time are the two important characteristics of DT, IDFA does not have the highest convergence and diversity, but its extreme performance in computing time is crucial for the application of DT in rescheduling.

**Figure 14:**
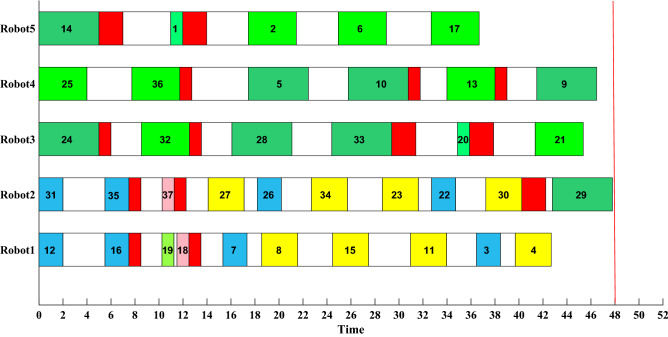
Initial scheduling plan.

Based on the result of IDFA in Table 7, the initial RAL task scheduling plan is shown in Fig. 14. The numbers in the rectangles represent the number of assembly tasks, ranging from 1 to 37, corresponding to 37 parts. The white rectangle represents the robot’s movement between different assembly points, the red rectangle represents the robot’s behavior of switching tools, and the same color represents the same type of parts. The cycle time of RAL is 48 s. Table 8 shows the parameters of the target threshold.

**Table 8:** Parameters of the target threshold.

Parameters	Value
$T_{sDT}$	230.8932
$L_{DT}$	7.3251
$\Delta T_{s0}$	2
$\Delta L_{0}$	0.5

**Figure 15:**
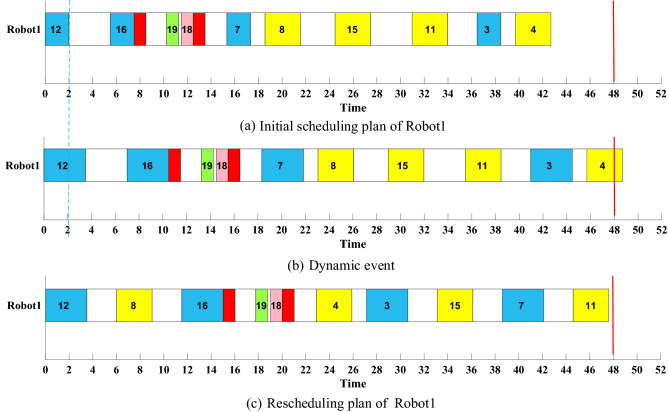
Unit level rescheduling.

Here we use simulated dynamic events and the initial state at the rescheduling point can be obtained by giving a random time T. As shown in Fig. 15, after the task numbered 12 is assembled, the processing time for Robot1 to assemble this type of part, or the basic assembly time, is extended by 1.5 s, as shown by the dotted line in Fig. 15b. At this point, if not rescheduled, Robot1 cannot complete the assigned tasks within the cycle time. Also, the total working time will not meet the requirement of the threshold. Therefore, the unit-level rescheduling strategy will be taken. The assembly tasks assigned to Robot1 will be adjusted first. If the requirements can be met, the rescheduling of the whole RAL will not be triggered. The rescheduling plan of Robot1 is shown in Fig. 15c. The performance of DT-based scheduling mechanism and scheduling mechanism without DT is shown in Table 9.

**Table 9:** Performance analysis and comparison of different scheduling mechanisms.

	$f_{1}$(s)	$f_{2}$(s)
Objectives	< 232.8932	< 7.8251
DT-based scheduling mechanism	232.3642	4.2862
Scheduling mechanism without DT	233.3279	6.0928

In Fig. 15, the assembly task order of Robot1 has changed, the priority of parts 8, 3 and 4 is advanced, while part 7 is delayed. The working time of Robot1 is 47.6065 s, still within the cycle time of RAL, and the time to obtain the rescheduling plan is 0.1782s.

The range of objectives in Table 9 is obtained based on the adaptive target threshold considering virtual data in VE and the initial scheduling plan before disturbance. The rescheduling plan based on DT has lower scheduling objectives than the one without DT, and satisfies the target threshold constraints.

**Figure 16:**
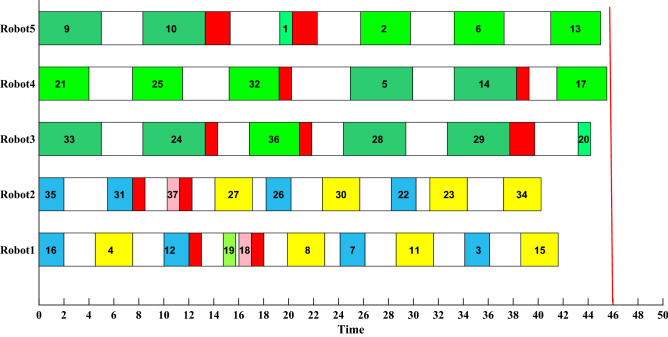
Production line level rescheduling.

In RAL assembly process, if the user places new requirements on the order, such as advance delivery, the cycle time of RAL must be adjusted as it determines the average time to produce a single product. For this user demand, the production line-level rescheduling is taken, a new assembly sequence is generated, and the assembly task assignment of the whole RAL has changed, as shown in Fig. 16. The cycle time of RAL is adjusted as 46 s, the total working time $f_{1}$ is 216.5573 s, and the load balancing $f_{2}$ is 7.5506 s, and the time to obtain the rescheduling plan is 0.9678 s.

## Conclusions

In this paper, a DT model consisting of PE, VE, and virtual-reality interaction and a digital twin-based RAL task scheduling framework are proposed. The PLC and RFID sensing technology is used to realize the perception and access of multi-source data. The VE is constructed by integrating geometric model, physical model, behavior model and rule model to precisely map the PE. Furthermore, the virtual-reality interaction mechanism promotes the evolution of the DT. Then a mathematical model is established by taking the total working time and the load balancing as the objectives. By analyzing the adaptive target thresholds from the perspectives of event trigger and user demand trigger, a DT-driven multi-level rescheduling strategy is proposed. At the same time, taking both the computing time and solution quality into consideration, an improved selection strategy and adaptive sparks number are introduced to a rescheduling algorithm for RAL based on an improved discrete fireworks algorithm. The real-time capability of the proposed algorithm are verified by task scheduling case study of RAL. In this work, other dynamic events, such as robot fault, are not considered in the task rescheduling. It is worth to consider them in the assembly task rescheduling.

## Data Availability

The datasets used and/or analysed during the current study available from the corresponding author on reasonable request.
